# Isometric Handgrip Training and Cardiovascular Risk Modulation: State of the Art

**DOI:** 10.3390/jcdd13040154

**Published:** 2026-03-30

**Authors:** Calogero Geraci, Francesca La Rocca, Salvatore Massimo Petrina, Agostino Buonauro, Giulio Geraci, Valentina Morello, Roberta Esposito

**Affiliations:** 1 Cardiobesity Group, Cardiology Unit, Azienda Sanitaria Provinciale of Caltanissetta (ASP), 93100 Caltanissetta, Italy; cgeraci81@gmail.com; 2 Department of Clinical and Experimental Medicine, University of Catania, 95122 Catania, Italy; 3 Cardiology Unit, Azienda Sanitaria Provinciale of Ragusa (ASP), 97100 Ragusa, Italy; 4 Santa Maria Della Pietà Hospital, ASL Napoli 3 Sud, Nola, 80035 Naples, Italy; 5 Department of Medicine and Surgery, “Kore” University of Enna, 94100 Enna, Italy; giulio.geraci@unikore.it; 6 Community Nutrition Biologist, 93100 Caltanissetta, Italy; 7 Department of Clinical Medicine and Surgery, Federico II University Hospital, 80131 Naples, Italy

**Keywords:** isometric handgrip, hypertension, cardiovascular prevention, vascular health, exercise prescription, frailty, cancer survivorship

## Abstract

Cardiometabolic diseases remain the leading cause of morbidity and mortality worldwide, despite major advances in pharmacological and lifestyle interventions. Exercise training is a cornerstone of prevention and treatment; however, adherence to traditional aerobic programs remains suboptimal. Isometric handgrip (IHG) training has emerged as a simple, time-efficient, and potentially effective strategy for improving cardiovascular and metabolic outcomes. This state-of-the-art review synthesizes current evidence on the physiological mechanisms underlying IHG training, including autonomic modulation, vascular function improvement, endothelial adaptation, and metabolic regulation. We summarize clinical data regarding its effects on blood pressure, arterial stiffness, endothelial function, insulin sensitivity, and inflammatory markers. Special attention is given to its applicability in specific populations, including hypertensive patients, individuals with metabolic syndrome, heart failure patients, and cancer survivors. We also discuss methodological heterogeneity across studies, safety considerations, and knowledge gaps. Finally, we outline future research directions needed to define optimal protocols and clarify long-term cardiometabolic benefits. IHG training represents a promising adjunctive strategy within cardiometabolic prevention and rehabilitation programs.

## 1. Introduction

Cardiometabolic diseases—including hypertension, obesity, impaired fasting glucose/type 2 diabetes, and atherosclerotic cardiovascular disease—cluster along a shared pathophysiological continuum driven by insulin resistance, chronic low-grade inflammation, endothelial dysfunction, autonomic imbalance, and adverse vascular remodeling. Lifestyle interventions remain the foundation of prevention and treatment across this spectrum, and structured exercise training is one of the most powerful non-pharmacological tools to improve cardiometabolic risk profiles and clinical cardiovascular outcomes. Contemporary evidence consistently supports exercise as a “multisystem therapy,” acting simultaneously on blood pressure, glycemic control, lipid metabolism, body composition, vascular function, and cardiorespiratory fitness, with downstream effects on morbidity and mortality [1].

Despite this strong rationale, real-world implementation remains challenging. Standard recommendations (e.g., accumulating ≥150 min/week of moderate-intensity aerobic activity plus resistance training) are effective but often limited by poor uptake and long-term adherence. Time constraints, low exercise self-efficacy, musculoskeletal limitations, and comorbidity burden disproportionately affect patients who stand to benefit the most—those with multiple cardiometabolic risk factors and established cardiovascular disease. Accordingly, “time-efficient” training paradigms that can be delivered at home, require minimal equipment, and still induce clinically meaningful cardiometabolic adaptations have become a major focus of preventive cardiology research and practice [1].

Within this landscape, isometric exercise training has re-emerged as a particularly attractive option. Isometric handgrip training (IHGT) is a simple, low-cost modality typically performed as brief bouts of sustained handgrip contractions at a prescribed intensity (commonly a percentage of maximal voluntary contraction), repeated several times per session, a few days per week. Several randomized controlled trials, later synthesized in meta-analyses, have demonstrated clinically meaningful reductions in resting systolic and diastolic blood pressure following IHGT, typically ranging from 5 to 10 mmHg after relatively short training periods, although early studies were limited by small sample sizes [2]. The signal of clinically relevant blood-pressure lowering, combined with an exceptionally low “time burden,” has positioned IHGT as a pragmatic candidate for patients with cardiometabolic risk who struggle to meet conventional exercise targets.

More recently, the broader field of isometric training has moved toward larger, more pragmatic effectiveness studies designed to address implementation barriers and generate scalable evidence in hypertensive populations. For example, the ISOFITTER randomized effectiveness study further reflects the growing interest in pragmatic home-based isometric prescriptions [3]. Although IHGT is distinct from other isometric modalities (e.g., wall squat), these parallel developments underscore an expanding interest in isometric prescriptions as feasible cardiometabolic interventions that can be self-administered, monitored, and potentially integrated with digital health strategies.

Given the escalating global burden of cardiometabolic disease, the persistent gap between guideline recommendations and patient adherence, and the need for accessible interventions that can be sustained over time, a state-of-the-art appraisal of cardiometabolic training with a specific focus on isometric handgrip exercise is timely. In this review, we will examine the physiological rationale for IHGT, summarize the current clinical evidence across cardiometabolic endpoints (with emphasis on blood pressure and vascular health), discuss practical prescription parameters and safety considerations, and identify key knowledge gaps and research priorities relevant to translation into routine care.

**Figure 1:**
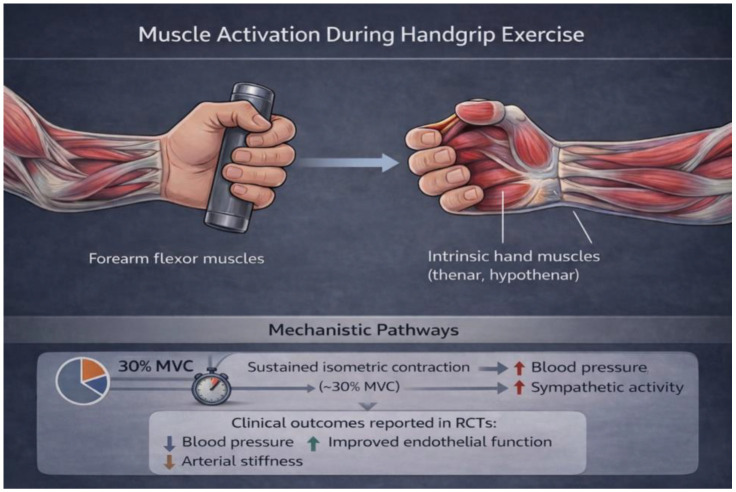
Conceptual representation of the multidomain physiological effects of isometric handgrip training (IHGT). Traditional handgrip devices primarily recruit forearm flexor muscles, whereas broader muscular engagement during isometric contraction may contribute to systemic autonomic activation, vascular shear stress modulation, and cardiometabolic signaling. The integrated response links peripheral muscle activation to central hemodynamic and metabolic adaptations relevant to cardiometabolic health.

A conceptual overview of the multidomain physiological engagement induced by isometric handgrip training is illustrated in Figure 1, highlighting the integration of muscular activation, autonomic modulation, vascular adaptation, and systemic cardiometabolic responses.

Several narrative and systematic reviews have previously summarized the effects of isometric handgrip training (IHGT), primarily focusing on blood pressure reduction and vascular responses [4,5,6,7].

However, the present review aims to extend current knowledge by: (I) providing an updated synthesis of the most recent evidence; (II) exploring emerging and under-investigated clinical settings, including frailty, cancer survivorship, and heart failure; and (III) adopting a translational perspective that integrates vascular, functional, and clinical outcomes.

## 2. Physiological Basis of Isometric Handgrip Training (IHGT)

### 2.1. Acute Cardiovascular Control During Static Handgrip

Static (isometric) handgrip elicits a characteristic pressor response—a rapid rise in arterial pressure with concomitant changes in heart rate and peripheral vascular resistance—driven by the integration of (I) central command and (II) the exercise pressor reflex originating from contracting skeletal muscle. In classic microneurographic experiments in humans, sustained handgrip increased muscle sympathetic nerve activity (MSNA) and arterial pressure, and the design of these studies helped disentangle the contributions of central command versus chemically sensitive muscle afferents (metaboreflex) in shaping sympathetic outflow during static exercise [8].

At the onset of handgrip, sympathetic activation supports blood pressure maintenance through vasoconstriction in non-working vascular beds, while local metabolic vasodilation occurs within the active forearm muscle. This acute pattern provides the physiological “stimulus” that, when repeated over weeks, is thought to promote favorable adaptations in autonomic and vascular control systems.

### 2.2. Autonomic Adaptations with Training

A leading mechanistic hypothesis for the antihypertensive effects of IHGT is improved autonomic regulation at rest, characterized by reduced sympathetic vasoconstrictor tone and/or enhanced vagal modulation. In a controlled training study in older adults with hypertension, IHGT lowered resting blood pressure and was accompanied by changes consistent with altered autonomic control as assessed by heart rate variability and blood pressure variability indices [9,10,11].

In parallel, evidence from clinical trials indicates that training responses may depend on supervision and implementation fidelity. A randomized trial comparing supervised versus home-based isometric handgrip training in medicated hypertensive patients showed blood pressure reductions in the supervised arm, while several secondary physiological markers (including autonomic indices) did not change meaningfully across groups—highlighting both the complexity of autonomic endpoints and the importance of protocol execution [12].

Repeated isometric contractions induce a transient increase in blood pressure mediated by the exercise pressor reflex and sympathetic activation. Chronic exposure to this controlled hemodynamic stimulus may promote adaptive changes in autonomic balance and vascular regulation. As illustrated in Figure 1, isometric contraction does not merely activate isolated forearm musculature, but initiates a broader hemodynamic and neurovascular cascade linking skeletal muscle activation to central cardiovascular regulation.

### 2.3. Vascular and Endothelial Mechanisms

IHGT may also act through vascular adaptations, particularly at the level of endothelial function. A mechanistic trial in medicated hypertensive individuals reported that bilateral handgrip training improved flow-mediated dilation (FMD)—supporting enhanced endothelium-dependent vasodilator function as a plausible pathway contributing to blood pressure lowering [13].

Conceptually, repeated bouts of isometric contraction can generate episodic alterations in local blood flow and shear stress in conduit arteries supplying the active limb. Over time, these hemodynamic stimuli may improve nitric oxide bioavailability and reduce endothelial dysfunction, thereby decreasing peripheral resistance and attenuating resting blood pressure.

### 2.4. Arterial Stiffness and Wave Reflection

Beyond endothelial function, IHGT has been linked to improvements in arterial stiffness and wave reflection, mechanisms that are highly relevant to cardiometabolic risk because they influence central (aortic) pressure and left ventricular afterload. In older adults, isometric handgrip training decreased indices of arterial stiffness and wave reflection; these hemodynamic changes were also associated with functional outcomes in that study population [14].

The magnitude of stiffness-related adaptations following IHGT may be influenced by baseline vascular characteristics and by intervention-related factors, such as training intensity, duration, and adherence, although direct evidence remains limited and findings are not entirely consistent.

### 2.5. Inflammation and Other Cardiometabolic Pathways: Current Evidence and Gaps

Acute isometric handgrip exercise can modulate circulating inflammatory mediators, and training intensity and duration appear to influence cytokine responses in experimental settings [13]. However, evidence from chronic IHGT interventions remains limited and heterogeneous. For example, small-scale studies have reported modest improvements in endothelial function and autonomic regulation, but data specifically linking IHGT to sustained changes in systemic inflammation, insulin sensitivity, or lipid metabolism are scarce. These domains therefore represent important priorities for future cardiometabolic-focused trials.

### 2.6. Practical Implication of Mechanistic Heterogeneity

Taken together, current mechanistic evidence supports IHGT as a multidomain stimulus—engaging autonomic control, endothelial function, and arterial hemodynamics—yet the relative contribution of each pathway likely varies by patient phenotype. The effectiveness of IHGT may be influenced by how the intervention is delivered, including factors such as contraction intensity, session duration, frequency, and supervision, although direct comparative evidence remains limited [10]. This has direct implications for clinical translation and may explain variability in blood pressure response across trials, including differences between supervised and unsupervised protocols [12].

## 3. Clinical Evidence

### 3.1. Hypertension

Hypertension is the most extensively studied clinical setting for isometric handgrip training (IHGT). Early randomized controlled trials demonstrated clinically meaningful reductions in resting systolic and diastolic blood pressure after short-term IHGT protocols (typically 6–10 weeks at ~30% maximal voluntary contraction, 3 sessions/week). In a landmark randomized trial, IHGT significantly reduced resting systolic blood pressure in hypertensive patients, supporting its potential as a non-pharmacological adjunct therapy [13].

A subsequent meta-analysis of randomized controlled trials confirmed that IHGT produces significant reductions in resting systolic and diastolic blood pressure, with magnitudes comparable to those observed with traditional aerobic training in some cohorts [2]. More recent pooled analyses have reinforced these findings, suggesting that isometric resistance training—including handgrip—elicits consistent antihypertensive effects across age groups and baseline blood pressure levels [14].

Importantly, effectiveness may depend on supervision and adherence. In medicated hypertensive individuals, supervised IHGT was associated with significant blood pressure reductions, whereas home-based unsupervised protocols showed more variable responses [15]. These findings highlight the role of implementation fidelity in clinical outcomes.

### 3.2. Arterial Stiffness and Vascular Function

Beyond peripheral blood pressure, several studies have investigated vascular endpoints. IHGT has been associated with improvements in endothelial function, as assessed by flow-mediated dilation (FMD), in hypertensive patients [16]. Additionally, reductions in arterial stiffness and wave reflection indices have been reported in older adults following isometric handgrip training, suggesting favorable effects on central hemodynamics [17].

These vascular adaptations may contribute mechanistically to blood pressure lowering and potentially to improved cardiovascular risk profiles, although hard cardiovascular endpoints have not yet been evaluated.

### 3.3. Heart Failure

Evidence specifically evaluating chronic isometric handgrip training (IHGT) as a therapeutic intervention in patients with heart failure (HF) is currently lacking. To date, no adequately powered randomized controlled trials have assessed the long-term effects of IHGT on functional capacity, ventricular remodeling, or clinical outcomes in either heart failure with reduced or preserved ejection fraction.

Isometric handgrip exercise has been employed primarily as a physiological stressor to investigate neurohumoral and hemodynamic responses in HF populations. Acute isometric contractions are known to increase systolic blood pressure and sympathetic activity via activation of central command and the muscle metaboreflex. While such responses have been studied under controlled laboratory conditions, these investigations do not provide evidence supporting chronic IHGT as a therapeutic modality in heart failure.

In contrast, structured aerobic and dynamic resistance exercise training is well established as safe and beneficial in clinically stable HF patients, improving exercise capacity and quality of life. However, these findings cannot be directly extrapolated to isometric handgrip training, which produces a distinct acute pressor response and different peripheral loading characteristics.

Given the absence of dedicated interventional trials, IHGT should currently be considered experimental in HF populations. Carefully designed randomized studies are required before routine implementation can be recommended.

In heart failure with preserved ejection fraction (HFpEF), a condition in which exercise intolerance is closely linked to vascular and peripheral limitations, and characterized by increased arterial stiffness, impaired ventricular–vascular coupling, and autonomic dysfunction, IHGT may have potential relevance through its effects on vascular function and blood pressure. Improvements in arterial stiffness and endothelial function observed in other populations suggest that IHGT could theoretically contribute to reducing afterload and improving hemodynamic efficiency in HFpEF.

However, direct evidence in HFpEF populations is currently lacking, and no studies have specifically evaluated the effects of IHGT on functional capacity, diastolic function, or clinical outcomes in this setting. To date, there are also no studies specifically evaluating the effects of IHGT on cardiac biomarkers, such as natriuretic peptides or cardiac troponins. Further research is needed to determine whether IHGT may represent a feasible adjunctive strategy in HFpEF management and whether these physiological adaptations translate into measurable changes in cardiac biomarker profiles.

### 3.4. Metabolic Endpoints and Observational Evidence Linking Handgrip Strength to Cardiometabolic Risk

Compared with hypertension, evidence for metabolic endpoints in response to isometric handgrip training (IHGT) is considerably less developed. While resistance training in general is known to improve insulin sensitivity and glycemic control, specific interventional data on IHGT remain sparse. The most comprehensive meta-analysis of IHGT to date primarily evaluated its blood pressure–lowering effects and did not assess metabolic outcomes such as insulin sensitivity, HbA1c, lipid profiles, or body composition changes [2]. To date, no large-scale randomized controlled trials have demonstrated sustained improvements in glycemic control, lipid metabolism, or adiposity attributable solely to IHGT, highlighting a significant evidence gap in this domain.

However, indirect support for the metabolic relevance of muscular function derives from observational studies examining handgrip strength (HGS) as a functional biomarker. A recent systematic review and meta-analysis including more than 40,000 participants demonstrated that lower relative HGS (normalized to body weight or BMI) is significantly associated with a higher prevalence of metabolic syndrome across diverse populations [18]. Importantly, relative HGS appeared to be a stronger predictor of metabolic risk than absolute strength values.

Similarly, prospective cohort studies and pooled analyses indicate that reduced handgrip strength is associated with an increased risk of incident type 2 diabetes mellitus. In a large cohort study with meta-analytic confirmation, relative HGS was inversely associated with future development of type 2 diabetes. Several confounding factors may influence the interpretation of IHGT studies, including antihypertensive medication use, baseline blood pressure, adherence to the intervention, variability in training protocols (e.g., contraction intensity, session duration, and frequency), and participant characteristics such as age, comorbidities, and baseline cardiovascular status. These factors likely contribute to the heterogeneity observed across studies and should be taken into account when interpreting findings [19]. A separate systematic review and meta-analysis of observational cohort studies further confirmed that greater handgrip strength is associated with a lower risk of developing type 2 diabetes [20].

Taken together, although direct evidence supporting IHGT-induced metabolic improvements is currently insufficient, observational data consistently identify handgrip strength as a robust marker of metabolic health and cardiometabolic risk. These findings provide a biologically plausible rationale for further investigating whether structured IHGT may influence metabolic pathways, including insulin sensitivity, inflammatory regulation, and body composition, in future adequately powered interventional trials.

To date, no studies have specifically evaluated the effects of IHGT in patients receiving GLP-1 receptor agonists. Given the established cardiometabolic benefits of these agents, future research should investigate whether IHGT may provide additive or complementary effects on blood pressure, vascular function, body composition, and overall cardiometabolic risk [21].

### 3.5. Use of Isometric Handgrip Training in Frail and Vulnerable Older Adults

Frailty is a geriatric syndrome characterised by decreased physiological reserve and increased vulnerability to stressors, and it is associated with adverse outcomes including falls, disability, and mortality. Skeletal muscle strength, particularly handgrip strength, is a core component of frailty assessment and a robust marker of overall physical function in older adults. Declines in handgrip strength are consistently associated with frailty status and related adverse health indicators, making it a valuable tool for screening and monitoring in this population [22].

Reduced handgrip strength has consistently been associated with frailty status, disability, and increased mortality risk [23,24].

While specific randomized controlled trials evaluating chronic isometric handgrip training (IHGT) in frail individuals are currently lacking, resistance-based exercise interventions in frail older adults have demonstrated improvements in physical performance, muscle strength, and functional capacity [25]. Given that IHGT is a low-load, time-efficient modality requiring minimal mobility and equipment, it may represent a feasible intervention in selected frail populations. However, evidence in this area remains limited but suggests potential benefits. For example, some studies have reported improvements in vascular function and autonomic regulation following IHGT interventions, although findings are heterogeneous and based on relatively small sample sizes [16].

These hypotheses remain preliminary and should be interpreted with caution given the absence of dedicated randomized controlled trials in frail populations. Nevertheless, they provide available data suggesting that IHGT may improve vascular function and autonomic regulation, as demonstrated by improvements in flow-mediated dilation and blood pressure reported in recent studies [16]. This biological plausibility is supported by the broader role of strength training and neuromuscular conditioning in maintaining functional capacity, reducing fatigue, and mitigating age-related decline [22].

Despite these findings, including modest reductions in blood pressure and improvements in vascular function reported in previous studies, the overall evidence base remains limited and heterogeneous [16]. Larger, longer-term trials are needed to determine whether IHGT can translate into meaningful clinical outcomes in frail populations, such as improvements in physical performance, reduction in fall risk, enhanced independence in activities of daily living, or slowed progression of frailty. Integration of IHGT into multimodal frailty management programs—potentially in combination with aerobic or balance training—may be a promising avenue for future research.

### 3.6. Cancer Survivors: A Cardio-Metabolic Lens (And Where IHGT Could Fit)

Cancer survivorship increasingly intersects with cardiometabolic medicine: many survivors carry a persistent burden of hypertension, dyslipidemia, insulin resistance, and treatment-related cardiovascular vulnerability that can outlast the oncology timeline. Across tumor types, structured aerobic and resistance exercise programs consistently improve patient-centered and functional outcomes—most reproducibly quality of life, cancer-related fatigue, body composition, and functional capacity—supporting exercise as a core pillar of long-term survivorship care rather than an “adjunct” intervention [26]. In breast cancer survivors, higher habitual physical activity after diagnosis has also been associated with lower all-cause mortality in prospective observational data, reinforcing the concept that movement behaviors remain clinically meaningful even when classical oncologic endpoints are harder to shift [27].

From a cardio-oncology perspective, the emerging question is no longer whether exercise is beneficial, but how to scale the right dose to the right survivor—including those with low reserve, cardiotoxicity risk, or barriers to supervised programs. Contemporary trials are actively testing pragmatic delivery models (e.g., remotely supported vs. partly supervised training) while tracking blood pressure and fitness, underscoring feasibility and implementation as decisive next steps [28]. Mechanistically oriented randomized work during trastuzumab therapy further suggests that exercise can engage cardiovascular-relevant signaling pathways (e.g., NRG1/HER axis), even if links to LVEF or VO_2_ changes remain complex and not yet linear [29]. In this landscape, isometric handgrip training (IHGT) is attractive as a minimal-equipment, low-time, potentially scalable stimulus that could be layered onto multimodal exercise prescriptions—particularly for survivors who are deconditioned, hypertensive, frail, or transitioning from supervised rehab to home-based maintenance. The opportunity (and the gap) is to move beyond blood pressure alone and test IHGT-informed strategies against a broader cardiometabolic survivorship phenotype, using rigorous endpoints (autonomic function, vascular health, insulin sensitivity, and imaging-based cardiac reserve) within cardio-oncology trial designs.

## 4. Practical Implementation

### 4.1. Protocols Used in Studies

Across the IHGT literature, the “classic” prescription is remarkably consistent: 4 × 2 min isometric contractions at ~30% of maximal voluntary contraction (MVC), typically with ~1 min rest between bouts and performed 3 sessions/week for 8–12 weeks. This core protocol has been implemented in medicated hypertensive cohorts and has served as the backbone for much of the mechanistic and clinical evidence base [13].

From a practical standpoint, how the 30% MVC target is operationalized matters:

- MVC assessment and calibration: Trials commonly set intensity as a percentage of a baseline MVC measured with a dynamometer. In real-world delivery, the key risk is “intensity drift” (patients progressively under-loading as strength adapts), arguing for periodic MVC reassessment or device-guided feedback where possible [12].
- Unilateral vs. bilateral delivery: Both approaches appear in trials. McGowan et al. explicitly tested unilateral vs. bilateral paradigms within the standard dose framework (4 × 2 min at 30% MVC, 3×/week, 8 weeks) [13].
- Program duration: Most controlled studies are 8–12 weeks, which is long enough to detect BP and vascular changes in responsive phenotypes, but still leaves uncertainty about maintenance dosing beyond the initial intervention window [10].

### 4.2. Home-Based vs. Supervised Programs

IHGT is often positioned as an ideal home-based intervention (low cost, minimal space, seated execution). However, the strongest cautionary signal for implementation is that supervision can meaningfully influence effectiveness, even when the nominal prescription is identical.

In a randomized trial in medicated hypertensive patients, supervised IHGT lowered brachial and central BP, whereas home-based IHGT did not, despite both being prescribed as 4 × 2 min at 30% MVC, 3×/week. This divergence strongly suggests that fidelity (true intensity, bout timing, adherence) is not guaranteed outside supervised settings [10].

Implementation implication: a pragmatic model for clinical services may be “supervised onboarding” (device familiarization + intensity calibration + breathing coaching) followed by structured home delivery supported by check-ins and objective device logs when available [12].

Alternative isometric exercise modalities, such as wall-squat training, have also demonstrated blood pressure–lowering effects in hypertensive populations, supporting the broader role of isometric exercise in cardiovascular prevention.

### 4.3. Safety Profile

From an implementation lens, IHGT sits at an important intersection: it is time-efficient and low-impact, yet it evokes an acute pressor response characteristic of isometric contractions. For most populations studied under trial conditions (including older adults and treated hypertension), protocols at ~30% MVC have been executed without signals of major safety concerns in the published RCT experience [13]. Potential contraindications to IHGT include uncontrolled hypertension, unstable coronary artery disease, recent acute cardiovascular events, and conditions in which transient increases in blood pressure may pose a risk. Careful patient selection is therefore recommended. In higher-risk patients, initial supervised sessions may be advisable, particularly to assess blood pressure response and clinical tolerance during early sessions. In higher-risk patients, initial supervised sessions may be advisable to assess blood pressure response and clinical tolerance. This approach may help identify exaggerated pressor responses or symptoms suggestive of myocardial ischemia before transitioning to home-based training.

That said, translation should remain conservative in higher-risk phenotypes because the immediate BP rise can be clinically relevant. The most defensible approach is:

- Screening and stabilization first (especially if BP is uncontrolled or symptoms suggest unstable cardiovascular disease).
- Technique coaching to reduce unnecessary pressor load (e.g., avoid sustained breath-holding/straining).
- Early-session monitoring in higher-risk individuals before fully home-based transition [12].

### 4.4. Adherence and Feasibility

IHGT’s feasibility is one of its most attractive translational features: sessions are short, equipment is minimal, and the protocol is structurally simple (timed bouts at a fixed target intensity). Yet the supervised-vs-home RCT evidence underscores that feasibility alone does not ensure training fidelity, and that real-world adherence must be evaluated not just as “sessions completed,” but as sessions completed at the intended intensity and duration [12].

At the evidence-synthesis level, meta-analytic work confirms BP-lowering efficacy of isometric handgrip exercise overall, but also highlights heterogeneity across trials—an important reminder that implementation details (population, supervision, intensity control) are not peripheral, but central to reproducible outcomes [2].

## 5. Frailty and Low-Load Isometric Training: A Translational Opportunity

Building on the previous section, the following section specifically focuses on the potential translational role of IHGT in frailty. The role of isometric handgrip training (IHGT) in frailty remains largely unexplored. Frailty is characterized by reduced physiological reserve and increased vulnerability to stressors, with muscle weakness—often assessed by handgrip strength—representing a key component associated with adverse outcomes, including disability and mortality [23,24]. Given its low physical demand and minimal mobility requirements, IHGT may represent a feasible exercise modality for individuals with limited exercise tolerance or multimorbidity. In other populations, IHGT has been associated with improvements in blood pressure, vascular function, and autonomic regulation, suggesting potential mechanisms that could be relevant to frailty.

However, direct evidence in frail populations is currently lacking. No adequately powered randomized controlled trials have evaluated the effects of IHGT on frailty-related outcomes, such as physical performance, functional independence, or quality of life.

Future studies are therefore needed to determine whether IHGT can meaningfully contribute to frailty management, either as a standalone intervention or as part of multimodal programs including aerobic exercise, resistance training, and nutritional support.

## 6. Conclusions

In conclusion, isometric handgrip training (IHGT) represents a simple, low-cost, and accessible intervention with consistent evidence supporting its antihypertensive effects, particularly in hypertensive populations. Additional data suggest potential benefits on vascular function, arterial stiffness, and autonomic regulation, although findings remain heterogeneous.

Despite these promising effects, important knowledge gaps persist. Evidence regarding metabolic outcomes, heart failure, frailty, and cardio-oncology populations remains limited and is often extrapolated from non-specific resistance training paradigms. Moreover, the clinical effectiveness of IHGT appears to be influenced by key implementation factors, including supervision, training intensity, and adherence.

Importantly, this review extends previous literature by integrating recent evidence and by addressing emerging and underexplored clinical contexts, including frailty, cancer survivorship, and heart failure, from a translational perspective that encompasses vascular, functional, and clinical outcomes.

Future studies should move beyond short-term blood pressure reductions and focus on clinically meaningful endpoints, mechanistic insights, long-term sustainability, and real-world implementation strategies. Rather than replacing conventional exercise programs, IHGT may represent a complementary approach to enhance adherence and expand access to exercise-based cardiometabolic prevention.

## Data Availability

The original contributions presented in this study are included in the article. Further inquiries can be directed to the corresponding author.
